# Ovine Model for Basic Training in Ear Surgery

**DOI:** 10.7759/cureus.109324

**Published:** 2026-05-20

**Authors:** Mara Tănase, Mihai I Tănase, Marcel Cosgarea, Alma A Maniu, Violeta Necula, Mirela C Stamate, Cristina Blebea, Constantin Stan, Maximilian Dindelegan, Doinel G Rădeanu

**Affiliations:** 1 Department of Otolaryngology, Iuliu Hațieganu University of Medicine and Pharmacy, Cluj-Napoca, ROU; 2 Department of ENT, Universitatea "1 Decembrie 1918” din Alba Iulia, Alba Iulia, ROU; 3 Department of ENT, Iuliu Hațieganu University of Medicine and Pharmacy, Cluj-Napoca, ROU; 4 Medical-Pharmaceutical Research Centre, Universitatea "Dunărea de Jos” din Galați, Galati, ROU; 5 Department of Surgery – Practical Abilities, Iuliu Hațieganu University of Medicine and Pharmacy, Cluj-Napoca, ROU

**Keywords:** ear surgery, experimental animal model, otolaryngology education, sheep's head, surgical training model

## Abstract

Background and objectives: Surgical proficiency in otolaryngology requires repetitive practice to master the complex spatial relationships of the temporal bone. While human cadaveric specimens remain the gold standard, their increasing scarcity and ethical constraints necessitate the validation of alternative biological models for training. This study aims to establish the sheep head as a viable anatomical model for training in ear surgery (ES) through comprehensive anatomical examination and training-based assessment of participants' satisfaction.

Materials and methods: Participants were divided into three groups according to their prior experience in ES: resident, junior specialist, and senior specialist. A total of 21 participants were included. Each participant in the study was assigned to perform the designated procedures on a single sheep's head. Following the completion of the procedures, each participant was provided with an 11-item comprehensive satisfaction questionnaire with a scale attributed from 1 to 5 ("Totally Disagree" to "Completely Agree"). The normality of distribution was checked by applying the Shapiro-Wilk Test. The Kruskal-Wallis test was applied to compare the study group sentiment of agreement towards individual procedures.

Results: The results of the satisfaction questionnaire were analyzed using the Kruskal-Wallis test, which did not reveal statistically significant differences between the responses of the three groups for several of the questionnaire items (p > 0.05). The average satisfaction score for each group was as follows: resident group 4.09 ± 0.54, junior specialist group 4.00 ± 0.55, and senior specialist group 4.2 ± 0.77.

Conclusions: A sheep's head can be successfully used for learning and practicing manual skills and the use of instruments specific to ES.

## Introduction

Ear surgery (ES), a cornerstone of ear, nose, and throat (ENT) practice, necessitates a profound understanding of the intricate anatomy of the human ear and the acquisition of refined surgical skills [1]. Traditionally, the acquisition of such skills has relied heavily on hands-on training, often involving dissection of cadaveric heads. This approach provides an invaluable opportunity for trainees to familiarize themselves with the complexities of the temporal bone and its associated structures, allowing the development of meticulous precision and dexterity [2]. 

However, the availability of cadaveric specimens for surgical training is becoming increasingly limited due to a confluence of medical, ethical, and logistical constraints [3]. This scarcity has prompted the exploration of alternative training modalities, with a particular focus on anatomical models that faithfully replicate the human ear [4]. The sheep head has emerged as a promising candidate for an ES training model due to its relative abundance, cost-effectiveness, and anatomical similarities to the human ear [5]. 

The use of animal models in surgical education has a long and rich history, dating back to the pioneering work of Galen in ancient Greece. The sheep, in particular, has been widely employed in various surgical disciplines, including neurosurgery, ophthalmology, and plastic surgery, due to its manageable size, anatomical features, and ease of procurement [6,7]. 

This study undertakes a comprehensive evaluation of the sheep head as an ex vivo model for ES training [8]. Through meticulous anatomical dissection and a validated satisfaction questionnaire administered to participants with varying levels of surgical expertise, we aim to assess the efficacy of this model in facilitating the acquisition of essential surgical skills. Our findings hold the potential to significantly impact ES training paradigms, offering a sustainable and accessible pathway for the next generation of otolaryngologists, similar to receding studies in which ovine simulation models were successfully utilized [9,10].

## Materials and methods

Specimen procurement and study setting

Following ethical approval from the Institutional Review Board of the Iuliu Hațieganu University of Medicine and Pharmacy (Registration number AVZ74/24.03.2025), 21 fresh-frozen sheep heads of the Native Romanian Țurcana breed were obtained from a local abattoir. As these specimens were byproducts of the food industry already destined for human consumption and were not specifically sacrificed for this research, they fall under a standard exemption for specific veterinary clearance for research purposes. Nevertheless, all specimens were sourced from a regulated facility to ensure baseline sanitary standards. During all dissection procedures, participants adhered to strict safety protocols, including the use of complete personal protective equipment (PPE) kits to prevent potential infection spread.

The practical surgical simulation training and anatomical evaluations were conducted at the Training Laboratory, Iuliu Hațieganu University of Medicine and Pharmacy, Cluj-Napoca, Romania.

Anatomical dissection and imaging

**Figure 1:**
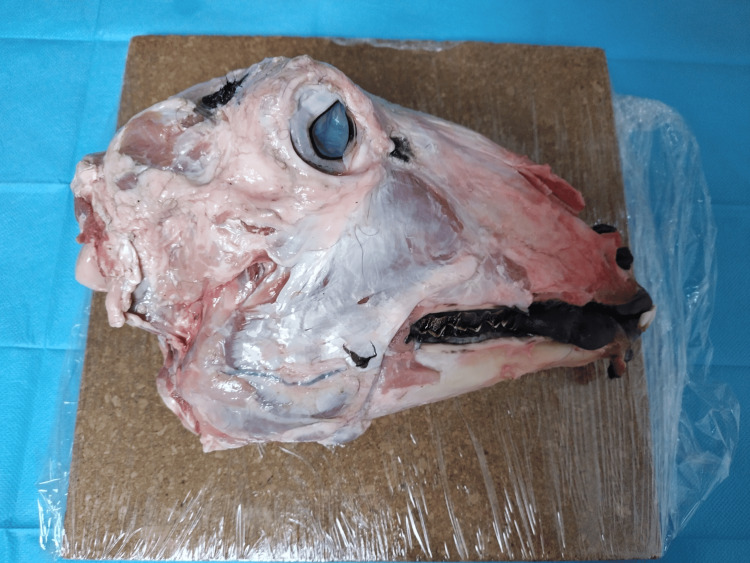
Sheep head prepared for dissection

**Figure 2:**
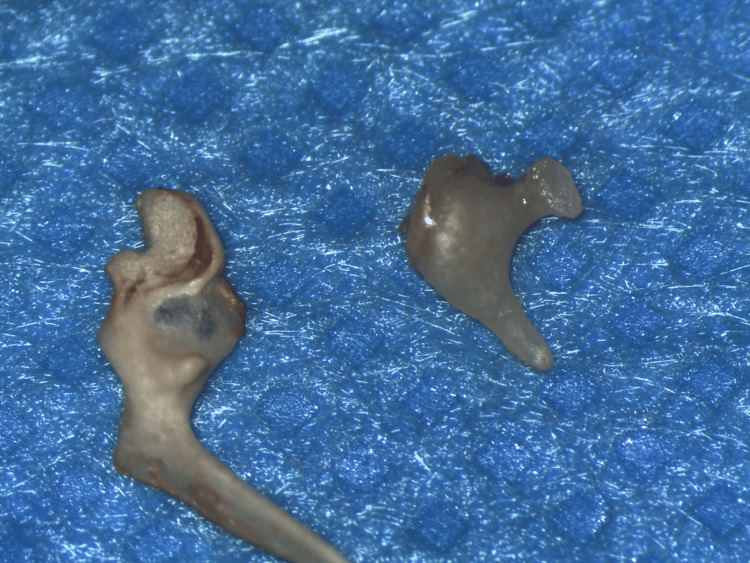
Middle ear ossicles after extraction

**Figure 3:**
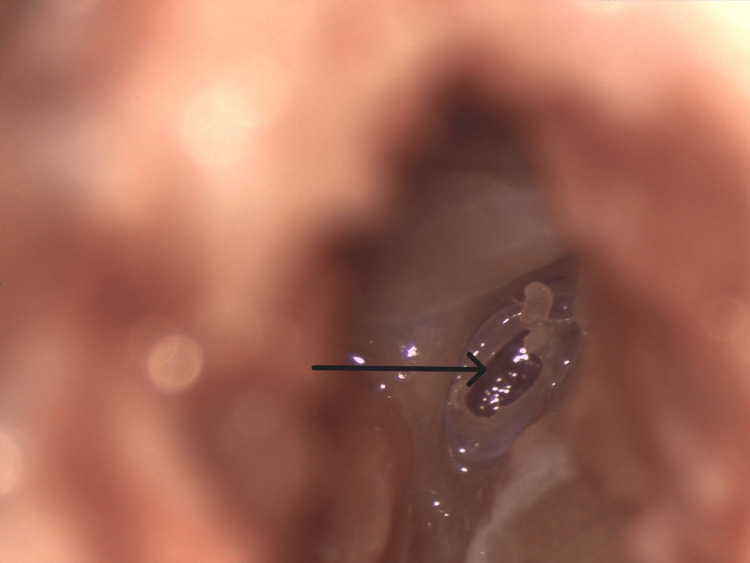
Microscopic view of the oval window indicated by the arrow

One sheep head was thawed at room temperature and subjected to detailed anatomical dissection after being sliced in half in the sagittal plane, during which photographs were taken as seen in Figures 1-3.

Model preparation

**Figure 4:**
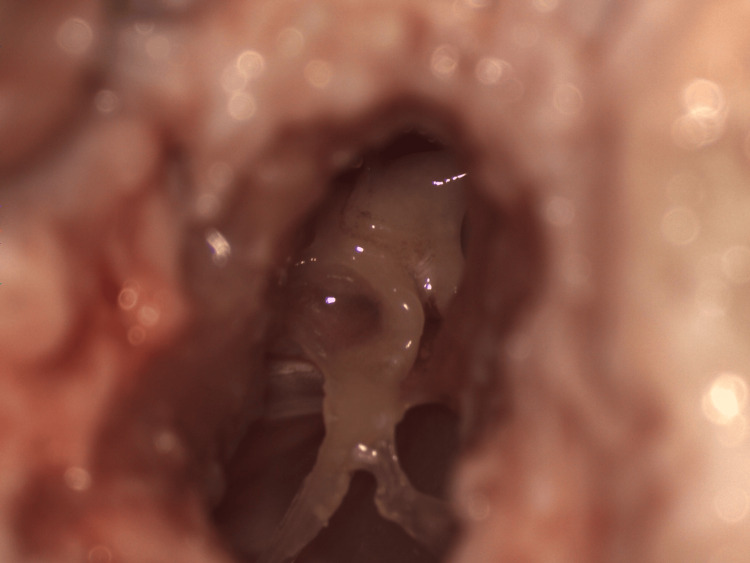
Microscopic view of the sheep's external auditory canal with a superiorly elevated tympanomeatal flap, providing exposure to the middle ear structures.

Prior to surgical maneuvers, the 21 frozen sheep heads were thawed at room temperature for approximately 14 hours. The external auditory canals (EAC) were then irrigated with a sterile saline solution, as seen in Figure 4.

Surgical instrumentation

A standard set of ES instruments was used for the training procedures, including an operating microscope, ear speculum, micro-instruments (picks, dissectors, scissors, forceps), suction apparatus, and a drill with otological burrs. It is important to choose the right burr type and size since this step improves both efficiency and safety during mastoidectomy.

Participant selection

Inclusion and Exclusion Criteria

We included active medical professionals currently training or practicing in otolaryngology who voluntarily consented to participate in the simulation workshop. Exclusion criteria included individuals without formal ENT surgical training or those unable to complete all designated procedural steps due to scheduling constraints.

Sample Size Calculation

Because this was designed as a pilot feasibility study utilizing a specialized, locally available cohort of regional trainees and specialists, a formal a priori sample size calculation was not performed. The final sample size (n=21) was determined entirely by total enrollment availability during the regional surgical simulation workshop cycle.

Stratification into Groups

Participants were stratified into three groups based on their prior experience in ES: (i) Resident group (10 otolaryngology residents with ES surgery experience in accordance with the UEMS (European Union of Medical Specialists) logbook for their respective study year), (ii) junior specialist group (six otolaryngology specialists with three to five years of experience in ES), and (iii) senior specialist group (five senior otolaryngologists with over 10 years of experience in ES).

Surgical procedures

Each participant performed a series of fundamental ES procedures on a single sheep head, encompassing both ears. The procedures performed by each participant included tympanomeatal flap elevation, myringoplasty, ossiculoplasty, and mastoidectomy. Additionally, a cochlear implantation simulation was conducted; however, as this was a simulated exercise focusing on the surgical approach and access to the round window rather than final electrode placement, no photographic record was maintained for this specific step. Participants' performance for this procedure was assessed based on the accuracy of the transmastoid exposure.

Satisfaction questionnaire

Following the completion of the surgical procedures, each participant was administered an 11-item satisfaction questionnaire (see Appendices). The questionnaire was developed by a panel of expert otolaryngologists and pilot-tested for clarity during the initial phase to ensure content validity regarding ear surgery competencies. While formal psychometric validation, such as Exploratory Factor Analysis or calculating Cronbach's alpha, was not performed, the design process focused on identifying key surgical steps and instrument handling requirements. Reliability was assessed through internal consistency checks among the expert panel to ensure the questions accurately captured the intended educational metrics.

Statistical analysis

Statistical analysis was performed using IBM SPSS Statistics for Windows, version 26.0 (IBM Corp., Armonk, New York, United States). Descriptive statistics were used to summarize the questionnaire data. The overall satisfaction score was calculated as the arithmetic mean of all 11 individual questionnaire items. While LS data is ordinal, results are reported as standard deviation (SD) to allow for comparison with existing surgical simulation literature and to provide a measure of central tendency across experience cohorts.

The normality of the data distribution was assessed using the Shapiro-Wilk test. The Kruskal-Wallis test was employed to compare the responses of the three participant groups, with results reported including the H statistic, degrees of freedom (df), and exact p-values. For all statistical analyses, a p-value (p) of less than 0.05 was considered to indicate statistical significance.

## Results

Descriptive anatomy of the sheep ear

The EAC of the sheep initially follows a horizontal trajectory before assuming a vertical, downward course. The tympanic membrane, analogous to its human counterpart, is composed of two distinct portions: the pars flaccida and the pars tensa. The pars flaccida intersects with the superior wall of the EAC at an obtuse angle, while the pars tensa forms an acute angle with the inferior wall. The epitympanum of the sheep ear houses structures largely comparable to those found in humans, including the head of the malleus, the incudomalleolar joint, the incus, the incudostapedial joint, the fossa incudis, the stapes, the oval window, the tympanic segment of the facial nerve, the pyramidal eminence, and the tendon of the stapedius muscle. 

While many structures are anatomically comparable, morphometric studies indicate that sheep ear structures are approximately two-thirds the size of their human counterparts. The malleus exhibits dimensions similar to the human malleus, though the handle is oriented more anteroinferiorly. The incus presents with notable variations, including a smaller body, shorter distance to the lenticular process, and arms of approximately equal length. While the stapes and oval window bear a close resemblance to human counterparts, the human stapes footplate is relatively larger, and the ossicular axes differ due to the sheep’s quadrupedal posture.

However, the facial nerve, situated superior to the oval window, is typically thicker and dehiscent (exposed) in the sheep. The hypotympanum is notably larger in the sheep. The round window, located anteroinferiorly, is accessible only through a transmastoid approach. The sheep mastoid is small, eburnated (dense), lacks an antrum, and its cells are filled with adipose tissue. 

Satisfaction questionnaire results

The Kruskal-Wallis test was employed to compare the responses of the three participant groups. No statistically significant differences were observed between the responses of the three participant groups (p > 0.05) for any of the questionnaire items, as shown in Table 1. 

**Table 1:** Participant satisfaction questionnaire results

Question	ENT Resident score, mean (SD)	ENT Junior score, mean (SD)	ENT Senior score, mean (SD)	H-Statistic (df =2)	p-value
Resemblance of anatomical structures to humans	3.1 (0.7)	3.2 (0.6)	3.0 (0)	0.117	0.943
Realistic perception of the tissues	4.7 (0.45)	4.4 (0.8)	4.33 (0.47)	1.904	0.386
Usefulness for improving hand-eye coordination	4.2 (0.6)	4.4 (0.48)	5.0 (0)	0.831	0.66
Applicability of basic ES instruments	4.1 (0.94)	4.4 (0.66)	4.0 (0)	1.489	0.475
Usefulness for acquiring basic microscopic examination skills	4.4 (0.48)	4.5 (0.5)	5.0 (0)	2.158	0.34
Usefulness for acquiring tympanomeatal flap elevation skills	4.8 (0.4)	4.5 (0.5)	4.66 (0.47)	1.343	0.511
Usefulness for acquiring myringoplasty skills	3.6 (0.8)	3.6 (0.48)	3.66 (0.47)	1.083	0.582
Usefulness for acquiring ossiculoplasty skills	3.0 (0.73)	2.6 (0.66)	2.2 (0.4)	3.311	0.191
Usefulness for acquiring mastoidectomy skills	4.8 (0.4)	4.2 (0.4)	4.66 (0.47)	1.083	0.582
Usefulness for acquiring cochlear implantation skills	3.8 (0.4)	4.4 (0.48)	4.66 (0.47)	4.861	0.088
Overall usefulness for surgical training	4.1 (0.53)	3.8 (0.6)	4.33 (0.47)	4.080	0.13

The Shapiro-Wilk test indicated that the data for several questionnaire items did not follow a normal distribution (p < 0.05), which further justified the use of the non-parametric Kruskal-Wallis test for group comparisons. The average satisfaction score for each group was as follows: resident group, 4.09 ± 0.54; junior specialist group, 4.00 ± 0.55; senior specialist group, 4.2 ± 0.77.

## Discussion

The findings of this study suggest that the sheep head is perceived as a high-fidelity anatomical model for training in ES based on participant satisfaction. With average satisfaction scores exceeding 4.0 across all experience levels, there is a clear professional consensus regarding its perceived usefulness. However, while the model was highly rated, these subjective results should be distinguished from objective training effectiveness; further studies are required to determine the model's impact on actual surgical skill acquisition in a clinical setting [11].

The primary strength of the sheep model lies in its ability to replicate the tactile feedback of live tissue, which is a critical component of surgical dexterity. Participants rated the realistic perception of tissues highly, with mean scores ranging from 4.33 to 4.7. This feedback is essential for learning the delicate balance of force required in the middle ear. However, it must be noted that fresh-freezing alters tissue properties. Post-thaw, the tympanic membrane may exhibit reduced elasticity, and canal skin may lack the natural mobility and turgor found in living human tissue. Furthermore, the absence of bleeding removes a critical layer of natural tissue feedback. Trainees should be explicitly taught that the living operating room environment will feel meaningfully different before transitioning from sheep models to human cases [12].

While the overall reception was positive, the lower scores for ossiculoplasty, ranging from 2.2 to 3.0, highlight important anatomical distinctions, particularly regarding the incus, which features a smaller body and shorter distance to the lenticular process compared to humans. Despite these differences, the model remains highly valuable for entry-level practice. It allows trainees to master the delicate handling of micro-instruments and the tactile nuances of middle ear manipulation in a three-dimensional space, providing a foundation of manual dexterity that is transferable to human reconstructive surgery even if some specific landmarks differ [13].

Conversely, procedures like myringoplasty and mastoidectomy were highly rated, with mastoidectomy receiving scores as high as 4.8. Despite the sheep mastoid being eburnated and lacking the pneumatized air cells characteristic of the human temporal bone, participants found it highly useful for mastering drill control. From a mechanical training perspective, the fresh-frozen sheep temporal bone provides acceptable cortical and cancellous drilling resistance. This makes the model particularly well-suited for high-repetition tasks before trainees progress to the more complex landmark identification. The sheep’s tympanic membrane features both a pars flaccida and a pars tensa, allowing trainees to practice tympanomeatal flap elevation with high anatomical relevance [14].

An interesting anatomical nuance identified in this study is the thicker and often dehiscent nature of the sheep's facial nerve, situated superior to the oval window. In a training context, this exposure acts as a built-in safety challenge, forcing the trainee to maintain constant microscopic awareness of the nerve's path. This "over-training" in a high-risk scenario is particularly beneficial when transitioning to clinical situations involving cholesteatoma or anatomical variations, as it fosters the heightened surgical caution required to avoid iatrogenic injury in the human operating room [15].

The logistical benefits of the sheep head model provide a compelling argument for its integration into global training programs. Human cadaveric specimens are becoming increasingly scarce due to medical, ethical, and legal constraints. The sheep model, being a byproduct of the food industry, offers a sustainable and abundant resource that bypasses many of the hurdles associated with human tissue procurement [16].

Cost-effectiveness remains a significant advantage, with specimens procured for approximately 4 euros (20 RON) each. However, it is important to distinguish between the low cost of consumables and the significant capital investment required for a functional laboratory. Establishing a dedicated workstation, including an operating microscope, micromotor drill, and microsurgery instruments, can range from $5,000 to $20,000 per station. While the sheep model democratizes access by reducing recurring costs, institutions must still account for these essential infrastructure expenses to ensure a high-quality training environment [17].

The use of the sheep head also addresses the ethical complexities surrounding surgical education. While cadaveric dissection is traditional, the ethical burden of sourcing and disposing of human remains can be significant. Utilizing an animal model that is already part of the agricultural supply chain (Țurcana breed) provides a more ethically accessible pathway for repetitive practice [18].

We must also consider the role of the sheep model in improving hand-eye coordination under the operating microscope. Senior specialists particularly noted its usefulness in this area, giving it a perfect mean score of 5.0. The ability to practice fine motor movements in the confined, three-dimensional space of the sheep’s middle ear is directly applicable to the challenges of human otological surgery [19].

The protocol of performing multiple procedures back-to-back also highlights the role of surgical fatigue. Research indicates that muscle fatigue rates in the deltoid and brachioradialis increase significantly after the first hour of operating, regardless of experience level. Beyond physical strain, cognitive fatigue can account for a significant portion of intraoperative errors. For complex procedures like mastoidectomy, where real-time anatomical decision-making is critical, the cognitive component is especially relevant. Future training curricula should consider these factors by implementing structured rest intervals between procedures to optimize skill acquisition [12].

Despite these advantages, the study's small sample size of 21 participants is a limitation that must be acknowledged. While the Kruskal-Wallis test showed no statistically significant differences between groups (p > 0.05), a larger cohort would be necessary to further validate these findings globally. Furthermore, this study focused primarily on participant satisfaction, a methodological baseline that aligns with established surgical simulation literature which distinguishes subjective trainee feedback from objective clinical skill-transfer metrics [20].

Future research should aim to quantify surgical performance improvements in the operating room following a curriculum of sheep head dissection. Measuring variables such as procedural time and complication rates in live cases would provide definitive evidence of the model's educational impact. This would bridge the gap between perceived utility and objective clinical proficiency [21].

## Conclusions

This study substantiates the use of the sheep head as a valuable and effective training tool for ES, demonstrating high participant satisfaction across all levels of surgical experience. The model's utility is particularly evident in procedures like myringoplasty and tympanomeatal flap elevation, which necessitate refined spatial awareness and dexterity within confined anatomical regions. Despite anatomical variations between sheep and human ears, especially in the mastoid region and the middle ear, the sheep head model provides a readily accessible and cost-effective platform for trainees to acquire and refine essential surgical skills in a controlled, risk-free environment. This model holds the potential to alleviate the increasing scarcity of cadaveric specimens, offering a sustainable alternative for surgical training.

Future research with expanded cohorts and objective performance evaluations is recommended to further assess the transferability of skills acquired on the sheep head model to live surgical scenarios. The integration of sheep head dissection into a multimodal ear surgery training curriculum promises to enhance surgical education. Future training pathways could benefit from a tiered, hybrid approach: using sheep temporal bones for entry-level drilling mechanics and high-repetition tasks, followed by virtual simulation and human cadaveric training for advanced anatomical landmark identification. Such a comprehensive framework will foster the development of a highly proficient surgical workforce, ultimately improving the safety and quality of patient care.

## Appendices

Participant satisfaction questionnaire

NAME SURNAME __________________________

DATE___________

We would like to learn more about your experience following the practice of otologic surgical maneuvers using the sheep head as an anatomical model, and we would appreciate it if you could rate your personal level of agreement with the following statements. There are no right or wrong answers; only you can provide us with this information. Thank you for your participation, and do not hesitate to ask for assistance if you find it necessary.

**Table 2:** Participant satisfaction questionnaire

Statement	Strongly disagree	Disagree	Undecided	Agree	Strongly agree
There is a good resemblance of anatomical structures	1	2	3	4	5
There is a realistic perception of the tissues	1	2	3	4	5
There is a good perception of depth	1	2	3	4	5
The model is useful for improving hand-eye coordination	1	2	3	4	5
There is good applicability of basic otosurgery instruments	1	2	3	4	5
The model is useful in acquiring basic skills for microscopic ear examination	1	2	3	4	5
The model is useful in acquiring basic skills for raising the tympanomeatal flap	1	2	3	4	5
The model is useful in acquiring basic skills for myringoplasty	1	2	3	4	5
The model is useful in acquiring basic skills for ossiculoplasty	1	2	3	4	5
The model is useful in acquiring basic skills for mastoidectomy	1	2	3	4	5
The model is useful in acquiring basic skills for cochlear implantation	1	2	3	4	5
The model is useful overall for surgical training	1	2	3	4	5
